# Clinical and Pathological Studies on Cattle Experimentally Infected with *Theileria annulata* in China

**DOI:** 10.3390/pathogens9090727

**Published:** 2020-09-03

**Authors:** Quanying Ma, Junlong Liu, Zhi Li, Quanjia Xiang, Jinming Wang, Aihong Liu, Youquan Li, Hong Yin, Guiquan Guan, Jianxun Luo

**Affiliations:** 1 State Key Laboratory of Veterinary Etiological Biology, Key Laboratory of Veterinary Parasitology of Gansu Province, Lanzhou Veterinary Research Institute, Chinese Academy of Agricultural Sciences, Xujiaping 1, Lanzhou 730046, China; maquanying2004236@126.com (Q.M.); liujunlong@caas.cn (J.L.); lizhi19880717@163.com (Z.L.); xiangquanjia2018@163.com (Q.X.); wjm0403@caas.cn (J.W.); liuaihong@caas.cn (A.L.); liyouquan@caas.cn (Y.L.); yinhong@caas.cn (H.Y.); 2 Jiangsu Co-Innovation Center for the Prevention and Control of Important Animal Infectious Disease and Zoonosis, Yangzhou University, Yangzhou 225009, China

**Keywords:** *Theileria annulata*, experimental infection, pathological changes

## Abstract

Theileriosis is an important tick-borne protozoosis that causes high morbidity and mortality in cattle. In this study, the pathological and clinical characteristics of cattle experimentally infected with *Theileria annulata* were investigated. The clinical findings revealed typical signs of bovine theileriosis, including fever, enlargement of superficial lymph nodes, anemia, and respiratory distress. The most common pathological features were petechial and ecchymotic hemorrhages on the mucosa and serosal surface, severe jaundice, pulmonary edema and emphysema, multifocal necrosis and numerous ulcerations in the abomasum, congestion and marble-like discoloration of the spleen, and severe intestinal ecchymotic hemorrhages. The main histological characteristics were proliferation and infiltration of lymphocytes, plasma cells, and macrophages in the lymph nodes, spleen, and lymph node mass. Macroschizonts were observed in the cytoplasm of lymphocytes and macrophages of the lymph nodes and spleen. This study has significance for basic research and the clinical detection and diagnosis of *Theileria annulata* infection and can aid the prevention and control of theileriosis and future studies of the pathogenic mechanisms.

## 1. Introduction

Tropical theileriosis is a tick-borne disease of cattle caused by *T. annulata*, which is transmitted by ticks of the genus *Hyalomma* [1]. The disease occurs worldwide, including in southern Europe, North Africa, and Central and East Asia [1,2,3]. In China, the disease mainly occurs in the arid and semiarid northern areas [4,5]. Due to the high mortality and morbidity of cattle infected with *T. annulata*, the disease results in significant economic losses and has impeded the development of cattle rearing [2,6].

In the natural environment, *T. annulata* requires two types of hosts to complete its life cycle. The first phase of its life cycle occurs in arthropod vectors of the genus *Hyalomma* and the second phase occurs in a mammalian host [7]. During tick feeding, the sporozoites of *T. annulata* are injected into the host and infect the various subsets of leukocytes and then develop into schizont stage in the host cells [8]. Then, the uninucleate merozoites are released into the bloodstream, invade erythrocytes, and develop into uninucleated piroplasms, and more than one piroplasms were observed in a red blood cell [9]. The diagnosis of tropical theileriosis was initially based on the clinical symptoms and microscopic examination of stained thin blood and lymph node smears [10]. In the case of subclinical or chronic infection of *Theileria annulata*, parasites might not be detected via these methods due to an extremely low parasitemia level [11]. The specific and sensitive molecular and serodiagnostic methods used to detect the parasite infection are PCR, LAMP, etc. [12,13,14,15,16,17]. The main clinical signs of tropical theileriosis include fever, slight nasal and ocular discharge, anorexia, salivation, enlargement of superficial lymph nodes, respiratory distress, acute anemia, jaundice, and death due to asphyxia [18,19,20]. The most common lesions were enlargement of the lymph nodes, enlarged spleen, pulmonary emphysema, and subcutaneous and intramuscular hemorrhages. Excessive pericardial and pleural fluids are evident, and the liver is larger than normal. Microscopy has revealed masses of proliferating lymphoid cells in the lymph nodes and spleen and the presence of Koch’s bodies in several organs [21,22]. Previous studies mainly focused on the clinical symptoms and pathology of *T. annulata* infection by natural infected water buffaloes and cattle [19,23,24], but systematic data for the clinical symptoms and pathology of cattle for artificially infected *T. annulata* is absent. In the present study, cattle were artificially infected with *T. annulata* sporozoites and the clinical symptoms and pathology changes were investigated. The results provide a better understanding of the pathogenesis and clinical diagnosis of this disease.

## 2. Results

### 2.1. Clinical Manifestation and Hematological Changes

**Figure 1:**
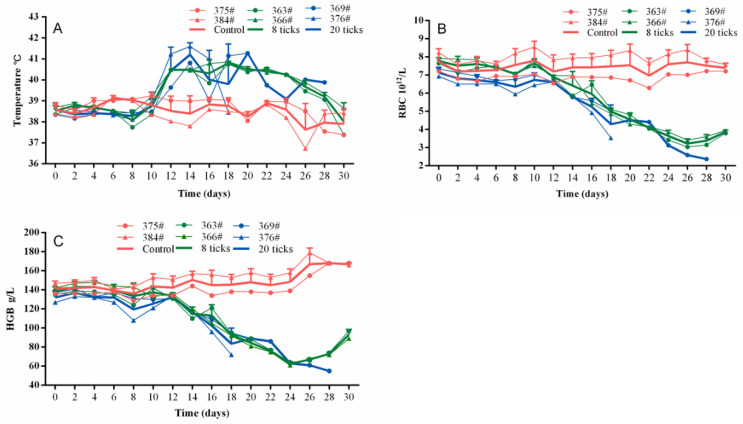
Physiological changes of cattle including control (No. 375, No. 384), 8 infective ticks (No. 363, No. 366), 20 infective ticks (No. 369, No. 376) for *T. annulata* sporozoite infection from the 0th dpi to the 30th dpi, including changes of body temperature (**A**), the numbers of red blood cells (**B**) and hemoglobin contents (**C**).

**Figure 2:**
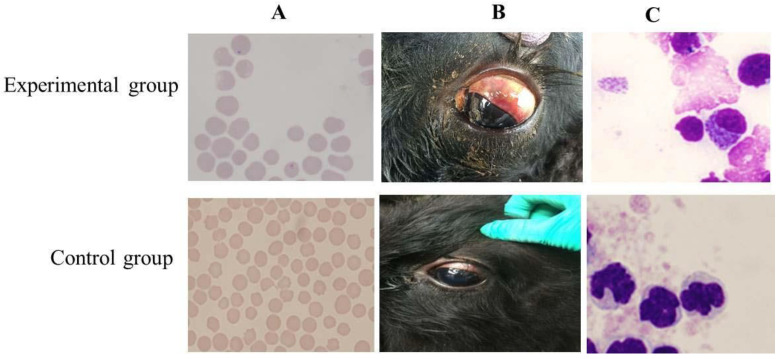
Clinical examination of the cattle infected with *T. annulata*. (**A**) piroplasms of *T. annulata* in red blood cells (1000×); (**B**) jaundice in the eye sclera; (**C**) microschizonts of *T. annulata* (1000×).

**Figure 3:**
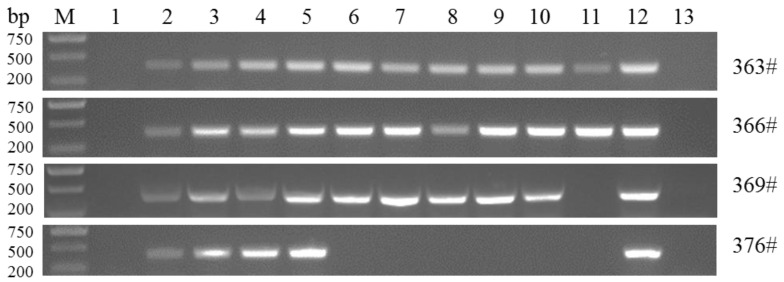
PCR detection of *T. annulata* infected cattle No. 363, No. 366, No. 369, and No. 376 every two days. Lane M: 2000 bp DNA marker; Lane 1-11: DNA from the 10th dpi to the 30th dpi of infected cattle; Lane 12: *T. annulata* positive DNA; Lane 13: *T. annulata* negative control.

The four infected calves demonstrated febrile responses from the 12th day post infection (dpi) and this lasted for 28 days for both (No. 363 and No. 366) and (No. 369 and No. 376). The maximum temperature of four calves reached higher than 40.5 °C compared with the control group (No. 375 and No. 384) (Figure 1A). Along with febrile responses, the piroplasms in erythrocytes (Figure 2A) were detected in Giemsa-stained blood smears on the 14th dpi, whereas the piroplasms of the control group were not examined. The maximum parasitemia was 12% and 70% when the corresponding infection dose was 8 ticks and 20 ticks, respectively. In addition, the genomic DNA of *T. annulata* (No. 363, 366, 369, 376) could be detected from the 10th to 30th dpi every two days (Figure 3). The genomic DNA of the control group were also detected and the results of the PCR were all negative. Compared to the control group, the calves showed clinical manifestations, such as angular, edema, and hemorrhage of the eyelid; jaundice in the sclera (Figure 2B); and enlargement of superficial lymph node, especially the anterior shoulder and inguinal lymph nodes. Moreover, the numbers of red blood cells (RBCs) were changed from 7.6 × 10^12^/L to 3.8 × 10^12^/L (No. 363), 7.9 × 10^12^/L to 3.9 × 10^12^/L (No. 366), 7.3 × 10^12^/L to 2.3 × 10^12^/L (No. 369), and 6.9 × 10^12^/L to 3.5 × 10^12^/L (No. 376) (Figure 1B). The hemoglobin concentrations (Hbs) were decreased from 135 g/L to 97 g/L (No. 363), 142 g/L to 89 g/L (No. 366), 137 g/L to 55 g/L (No. 369), and 127 g/L to 72 g/L (No. 376) (Figure 1C). The calf of No. 369 died on the 28th dpi with clinical symptoms of shortness of breath, rapid heartbeat, and extreme anemia. Cattle (No. 376) died on the 20th dpi.

### 2.2. Necropsy

**Figure 4:**
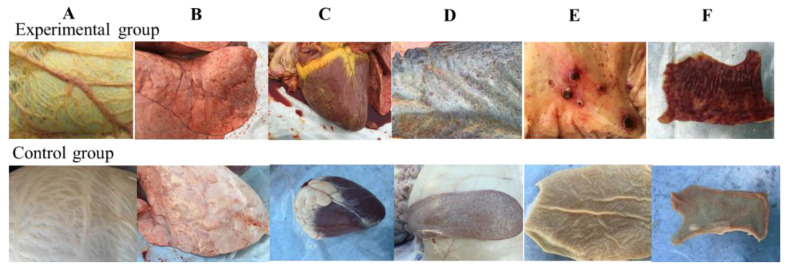
The pathological changes of the main organs of the cattle infected with *T. annulata*. (**A**) jaundice in the subcutaneous fat and omentum; (**B**) emphysema and carnification of pulmonary lobule and various hemorrhages in lung; (**C**) the pericardium was slightly enlarged and hemorrhagic and the coronal groove was severely jaundiced; (**D**) the spleen exhibited marble-like lesions and severe hemorrhages; (**E**) ulcers and multifocal necrotic lesions were observed in the abomasal mucous membrane; (**F**) hyperemia and hemorrhage throughout the whole intestinal tract.

The microschizonts of *T. annulata* could be detected in the lymph node smears (Figure 2C). Severe jaundice in the subcutaneous fat and omentum (Figure 4A), ecchymotic hemorrhages, and petechial in the mucosal and serosal surfaces were observed on necropsy. The endocardium and pericardium were slightly enlarged and hemorrhagic and the coronal groove was severely jaundiced (Figure 4C). Pulmonary edema and emphysema together with ecchymotic hemorrhage were observed on the lung surface, the lung lobule had become meaty (Figure 4B), and the section was enlarged and fluid filled. The spleen exhibited marble-like lesions and severe hemorrhages (Figure 4D). The liver and kidney were enlarged and displayed ecchymotic hemorrhagic on the surface. Hemorrhages, ulcers, and multifocal necrotic lesions were observed in the abomasal mucous membrane (Figure 4E). Most of the intestine of infected cattle exhibited necrosis and hemorrhage (Figure 4F) and there were large areas of mucosal surface petechial and ecchymotic hemorrhages and severe jaundice of the mesentery in the infected cattle.

### 2.3. Histological Characteristics

The most pronounced histological changes were the proliferation and infiltration of plasma cells, lymphocytes and macrophages in the spleen, lymph nodes, and Peyer’s patches. In other organs, various degrees of pathology were also observed. The histological changes of each organ were as follows.

**Figure 5:**
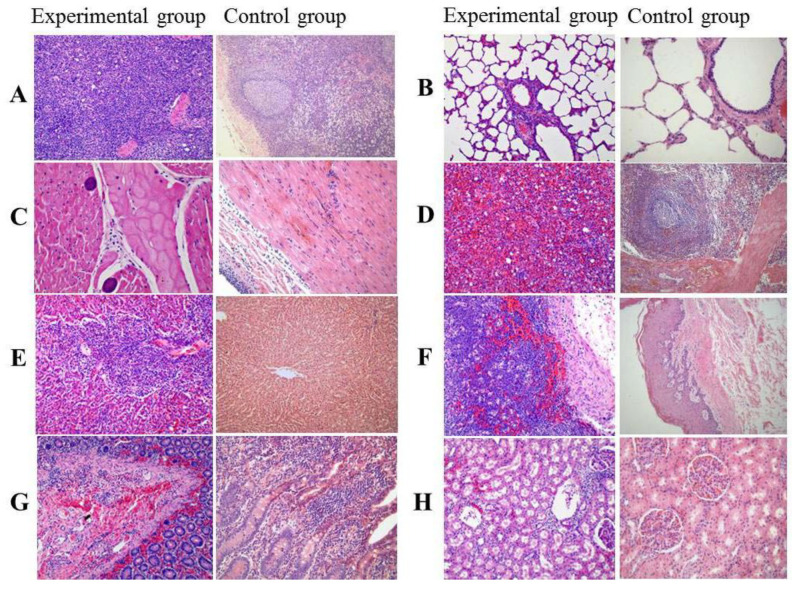
Histopathological changes of the main organs of cattle infected with *T. annulata* (200×). (**A**). Lymph nodes; (**B**). Lung; (**C**). Heart; (**D**). Spleen; (**E**). Liver; (**F**). Stomach; (**G**). Intestine; (**H**). Kidney.

Lymph nodes: hyperemia; hemorrhage, edema, necrosis of lymph node trabeculae; degenerative lesions; disappearance of lymph node structure; scattered necrosis of lymphocytes. Lymph sinus filled with a large number of cells, including red blood cells, neutrophils, and macrophages (Figure 5A).

Lung: alveolar expansion, emphysema in some parts, thickened alveolar septa, occasional venous vascular with a large amount of inflammatory substances and infiltration of lymphocytes, plasma cells, macrophages, and eosinophils around some large arteries (Figure 5B).

Heart: occasional hemorrhages; a small amount of infiltration of central granulocytes, monocytes, and lymphocytes in the hemorrhagic sites; some myocardial fibers with severe hemorrhage, and more nodular nodules (Figure 5C).

Spleen: vacuole-like structures in the white pulp; loss of splenic trabeculae and nodules; proliferation of lymphocytes, macrophages, and plasma cells; diffuse hyperemia; loss of the lymphatic sheaths around the arteries (Figure 5D).

Liver: large amounts of inflammatory exudates and necrotic foci on the surface; degeneration of hepatocyte granules; necrosis and fibrosis around the portal area and central vein; infiltration of lymphocytes, plasma cells, macrophages, and eosinophils; atrophy and obstruction of the bile ducts; large amount of bile accumulated in the liver sinusoids (Figure 5E).

Stomach: mucosal epithelial detachment, coagulative necrosis, and visible pigmentation in the necrotic area; diffuse lymphocytes, plasma cells, macrophages, and eosinophils in the mucosal layer; large hemorrhages in the lamina propria; submucosa sporadic hemorrhage; focal hemorrhage in the muscular layer; a large number of gastric glands replaced by inflammatory tissues (Figure 5F).

Intestine: infiltration of inflammatory cells in the mucosal layer; intestinal glands in some areas filled with red blood cells; mucosal necrosis, edema, epithelial detachment; partial loss of intestinal villi structure; diffuse hemorrhage of the lamina propria and submucosa accompanied by the infiltration of lymphocytes, macrophages, and plasma cells; loose local tissue; and hemorrhage separated from the muscle layers (Figure 5G).

Kidney: focal hemorrhage and necrosis in the renal cortex, glomerular edema, hyperplasia of endometrial cells and vascular endothelial cells, contraction or loss of the renal capsule, occasional protein exudates or foreign bodies in renal capsules, loss of glomerular lysis, accumulation of renal tubular and lymphatic reticulum cells, infiltration of some glomeruli and vessels, and protein tubular patterns in some renal tubular lumens (Figure 5H). Further, the scores of the main histopathological organs for the experimental and control groups of cattle are shown in Table 1.

## 3. Discussion

In the present study, the clinical signs and pathological changes of cattle experimentally infected with *T. annulata* sporozoites were investigated. The clinical features observed in the present study were similar to those observed in cattle naturally infected with *T. annulata* through tick bite in previous studies [3,21,24,25]. As shown in Figure 1, the body temperature, RBC numbers, and Hbs of cattle (No. 369 and No. 376) were more significantly changed than cattle (No. 363 and No. 366). The results in our study demonstrated that the cattle showed various degrees of severity of clinical manifestations when they were challenged with different doses mimicking infection via different numbers of ticks, i.e., 8 and 20 ticks. Therefore, the findings of our study re-demonstrated that the symptoms and severity of *T. annulata* infection were closely related to the dose of *T. annulata*. Of course, the tick and host type also play a vital role in the severity of *T. annulata* infection.

The temperatures of cattle were recorded every two days after inoculation with *T. annulata* in our study. The temperature increased following infection, whereas the levels of both RBC and Hbs decreased and severe anemia appeared in the late stage of infection due to the piroplasms in RBCs [26] or the autoimmune response [27]. In our study, the results of blood smear showed that the piroplasms and schizonts were both observed in the red blood cells and lymphocytes at the 14th dpi, respectively, in Figure 2. However, as shown in Figure 3, the result of PCR detection for *T. annulata* was also positive at the 12th dpi. A possible reason for this is that the PCR assay was more sensitive than blood smear.

In our study, based on the results of clinical and histopathological changes of *T. annulata* infection in Figure 4 and Figure 5, *T. annulata* infection caused necrotic lesions, including hepatomegaly, hemorrhage of the mucosa, and gastric ulcer. These lesions are similar to the lesions reported by Sandhu [28] that caused severe hepatobiliary dysfunction due to anemia and jaundice. Other organs also showed various degrees of lesions, especially pulmonary edema and emphysema; petechial and ecchymotic hemorrhages in the spleen, intestinal lining, and lymph gland; and large areas of jaundice in the epidermis, including omentum and mesentery. Infiltration of lymphocytes and macrophages was observed in the interstitial spaces surrounding the muscle (heart), the portal vein (liver), and the kidney. The results here are consistent with the previous report of *T. annulata*-infected cells undergoing transformation and proliferation in the hematopoietic system [29]. The proliferation and transformation of *T. anulata* infection are related to the casein kinase II, JNK signalling, HIF1alpha, etc. [9,30].

In conclusion, the pathological changes in cattle experimentally infected with *T. annulata* included mainly hemorrhage and necrosis of the liver, spleen, and intestine, as well as various degrees of hemorrhage, swelling, and nodule formation of other organs and necrosis of lymphocytes, reticuloendothelial cells, and other cells. The observed features of severe anemia, wasting, jaundice, and anterior shoulder and posterior bone lymphadenopathy can be used as a basis for the clinical diagnosis of *T. annulata* infection. The findings provide strong evidence for the epidemiological investigation and prevention of the disease in China, building the theoretical basis for future basic research on tropical theileriosis.

## 4. Materials and Methods

### 4.1. Parasites and Ticks

*T. annulata* parasites (Kashi strain) were obtained from the Vectors and Vector-borne diseases laboratory (VVBD Lab) of Lanzhou Veterinary Research Institute (LVRI) (Lanzhou, Gansu) [31]. Piroplasm-free ticks of *Hyalomma anatolicum antatolicum* were maintained in the VVBD Lab.

### 4.2. Preparation of Infected Ticks

*T. annulata*-free cattle that were detected by microscopic (Olympus, Tokyo, Japan) examination, PCR, and ELISA [32,33] were injected with *T. annulata*-infected red blood cells via the jugular vein. Then, 10 days post injection, unfed *Hy. anatolicum anatolicum* larvae were allowed to feed on the back of the cattle. The presence of *T. annulata* piroplasms was assessed daily by microscopic examination of stained blood smears made with venous blood from ears. Engorged *Hy. anatolicum anatolicum* nymphs were collected when the parasitemia level was higher than 5% and stored in an incubator at 28 °C under 60% humidity for molting.

### 4.3. Preparation of T. annulata Sporozoite Suspension

The sporozoite suspension of *T. annulata* was prepared using the previously reported methods with some modifications [34]. Unfed adult ticks infected with *T. annulata* were fed on the back of healthy cattle for 3 to 4 days. Then, the half-engorged adult ticks were removed from the cattle and collected into 50 mL centrifuge tubes. The collected ticks were rinsed 3–4 times with ddH_2_O to remove debris and hairs. The ticks were then washed with 70% ethanol followed by RPMI-1640 medium containing Hanks balanced salt solution and 2% Penicillin a total of 3 times. After washing, the ticks were immersed in 25 mL of RPMI-1640 medium containing 2% Penicillin and incubated on ice for 10 min. Then, they were transferred to a mortar and ground in 2–5 mL of RPMI-1640 medium containing 3.5% bovine serum albumin (BSA) and 2% Penicillin. Finally, the sporozoite suspension was transferred to a 50 mL tube and the total volume was adjusted to 4 ticks per 1 mL of sporozoite suspension, with RPMI-1640 medium containing 3.5% BSA. The sporozoite suspensions were then stored in liquid nitrogen until the next step.

### 4.4. Infection of Cattle Using T. annulata Sporozoite Suspension

Six calves (No. 375, No. 366, No.376, No. 384, No. 369, and No 363), 6-12 months old, were purchased from Yuzhong country of Gansu province, China and were detected with microscopic examination, PCR, and ELISA to confirm that they were free of piroplasma infection. The animals were maintained in a shielded cattle house and fed with tick-free food and water. Calves No. 363, No. 366 and No. 369, and No. 376 were inoculated subcutaneously each with 2 mL and 5 mL of sporozoite suspension, respectively, equivalent to 8 and 20 infective ticks. Calf No. 384 and No. 375 were injected with 5 mL RPMI-1640 medium as a control.

### 4.5. Clinical and Histopathological Examination

Blood was sampled every two days after infection for determining hematological indexes using Hematology Analyzer (CA-700 STAC^®^) and extracting genomic DNA. Thin blood smears were prepared with the veinous blood from the ears, fixed with absolute methanol, stained with Giemsa, and examined for the presence of *T. annulata* using light microscopy at ×1000 magnification. The rectal temperature and clinical symptoms were monitored every two days. The necropsy was performed for various organs when the animals showed serious clinical symptoms. Various tissues of sick and normal calves, including heart, liver, spleen, lungs, kidneys, abomasum, intestine, and lymph nodes, were collected for histopathological examination. The samples were fixed with 4% neutral formaldehyde, embedded in paraffin, stained with hematoxylin-eosin, and examined under a light microscope.

### 4.6. DNA Extraction and PCR Detection

Genomic DNA was extracted from the blood samples using a QIAamp DNA Mini Kit (QIAGEN, Hilden, Germany) according to the manufacturer’s manual. The infection of *T. annulata* was detected using a previously reported PCR method [32].

## Figures and Tables

**Table 1:** The scores of the main histopathological organs for the experimental and control groups of the cattle.

	Organs	Lymph Nodes	Lung	Heart	Spleen	Liver	Stomach	Intestine	Kidney
Scores									
Experimental		2	2	3	3	3	4	3	2
Control		0	0	1	0	1	0	1	0

Note: all histopathological changes of organs showed that the scores for 0, 1, 2, 3, 4 presented the degrees of histopathological changes on no lesions, slight, mild, moderate, and severe, respectively.
